# Intact skin and not stripped skin is crucial for the safety and efficacy of peanut epicutaneous immunotherapy (EPIT) in mice

**DOI:** 10.1186/2045-7022-2-22

**Published:** 2012-11-12

**Authors:** Lucie Mondoulet, Vincent Dioszeghy, Emilie Puteaux, Mélanie Ligouis, Véronique Dhelft, Franck Letourneur, Christophe Dupont, Pierre-Henri Benhamou

**Affiliations:** 1 DBV Technologies, Green Square, Bagneux, France; 2 Institut Cochin, Université Paris Descartes, INSERM-U567, Paris, France; 3 Hôpital Necker-Enfants Malades, Université Paris Descartes, Paris, France

**Keywords:** Food allergy, Immunotherapy, Epicutaneous, Peanut

## Abstract

**Background:**

Epicutaneous immunotherapy (EPIT) on intact skin with an epicutaneous delivery system has already been used in preclinical and clinical studies. In epicutaneous vaccination and immunotherapy, the stripping of skin before application of the allergen is suggested to facilitate the passage of allergen through immune cells.

**Objectives:**

The aim of this study was to compare the immunological response induced by EPIT performed on intact and stripped skin in a mouse model of peanut allergy.

**Methods:**

After oral sensitization with peanut and cholera toxin, BALB/c mice were epicutaneously treated using an epicutaneous delivery system (Viaskin® (DBV Technologies, Paris) applied either on intact skin or on stripped skin. Following EPIT, mice received an exclusive oral peanut regimen, aimed at triggering esophageal and jejunal lesions. We assessed eosinophil infiltration by histology, mRNA expression in the esophagus, antibody levels and peripheral T-cell response.

**Results:**

EPIT on intact skin significantly reduced Th2 immunological response (IgE response and splenocyte secretion of Th2 cytokines) as well as esophageal eosinophilia (2.7 ± 0.9, compared to Sham 19.9 ± 1.5, p < 0.01), mRNA expression of Th2 cytokines in tissue and intestinal villus sub-atrophia (2.9 ± 0.2 vs Sham, 2.1 ± 0.2, p < 0.05). By contrast, EPIT on stripped skin reinforced Th2 systemic immunological response as well as eosinophil infiltration (26.8 ± 15.1), mRNA expression of Th2 cytokines and duodenal villus/crypt-ratio (2.4 ± 0.3).

**Conclusions:**

Epicutaneous allergen-specific immunotherapy needs the integrity of superficial layers of the stratum corneum to warranty safety of treatment and to induce a tolerogenic profile of the immune response.

## Background

A new method of allergen-specific immunotherapy, via the epicutaneous route (epicutaneous immunotherapy, EPIT), is currently under investigation, using a unique epicutaneous delivery system (Viaskin®, DBV Technologies, Paris, France) consisting of a central transparent plastic membrane (11 mm in diameter) of polyethylene electrically charged with electrostatic forces and an adhesive sheath of nonwoven film. Dry powder of proteins is maintained on the backing by electrostatic forces. An occlusive chamber is created on the skin that rapidly generates moisture and releases the allergen from its support. The allergen is then absorbed by the skin where it interacts with epidermal immune cells [1]. EPIT consists of repeated and prolonged administrations of peanut protein extract on intact skin, allowing to reach the immune system without any risk of massive transcutaneous passage [2]. Some encouraging results in children severely allergic to cow’s milk [1] have been already published as well as several studies on mice sensitized to pollen, ovalbumin, house dust mites and peanuts [2-5]. The preclinical analysis of the different events occurring during EPIT with Viaskin® showed that after a prolonged application on intact skin, the allergen is taken up by dendritic cells in the superficial layers of the stratum corneum and transported, after internalization, to the draining lymph nodes, with variations according to the previous level of sensitization of the mice [2]. Contrary to stripped skin, when the Viaskin® is applied on healthy skin, the amount of allergen that passes freely through the skin is very limited and the passage of the allergen is mostly intracellular [2]. Also, recently, this action was shown to be powerful since it prevented the gastro-intestinal lesions induced by sustained oral exposure in sensitized mice [5]. Interestingly, Viaskin® acts through the application of the peanut protein extract on intact skin contrary to all other attempts of EPIT described to date. In epicutaneous vaccination [6,7] as well as in EPIT [8], authors suggest stripping the skin before application of the allergen in order to facilitate the passage through the skin immune system. The aim of the current study was to delineate the role of the skin preparation during EPIT in terms of both safety and efficacy.

## Methods

### Reagents and mice

Peanut protein extract (PPE) used for sensitization and immunotherapy was purchased from Greer laboratories (Lenoir, NE, USA). The endotoxin content of 100μg of peanut protein extract was evaluated below 50 EU (negligible values). Ara h 1 content in 500μg of PPE was estimated at 2.8% (ie. 14μg) using commercial ELISA kit (Indoor Biotechnologies, Charlottesville, VA, USA) according to the manufacturer’s instructions. Cholera toxin (CT) was purchased from List Biological Laboratories Inc. (Campbell, CA, USA).

Three-week-old female BALB/c mice (Charles River, Lyon, France) were purchased and housed under standard animal husbandry conditions. All experiments were performed according to the European Community rules on animal care and with permission 92-305 from the French Veterinary Services.

### Induction of peanut allergy, EPIT treatment and induction of esophageal and jejunal injuries

Twenty-four mice were first sensitized to peanut proteins by means of 6 intra-gastric gavages (D1, D7, D13, D19, D25, D32) as previously described [4], with 1 mg of PPE mixed with 10μg of CT. Then, 8 sensitized mice were treated by EPIT on intact skin (EPIT) and 8 other sensitized mice were treated on tape-stripped skin (stripping+EPIT). The last 8 sensitized mice were sham-treated (Sham) and received an empty Viaskin® (no protein administered on the skin). During sensitization and immunotherapy, all the mice were fed with standard mouse diet free of peanut proteins; the absence of low amounts of peanut protein was showed by a specific ELISA to Ara h 1 validated in food matrix (Indoor Biotechnologies, Charlottesville, VA, USA). After sensitization and immunotherapy periods, the animals were first orally challenge with high amounts of peanut proteins to measure histamine release in blood sample and then underwent sustained oral exposure to peanuts for 10 consecutive days as already published [5]. Eight naive mice serving as controls received the same procedures. The day after the last challenge, mice were anesthetized and sacrificed and sample studies were performed as described above.

### Epicutaneous immunotherapy (EPIT)

1/ Preparation of the skin and application of Viaskin®

Hair was removed from the back of mice under anesthesia using an electric clipper and depilatory cream without corticoid as already described [3,4]. Twenty-four hours later, after total recovery of the skin evaluated by Trans Epidermial Water Loss (TEWL) measurements, the Viaskin® loaded with 100μg PPE (EPIT and stripped+EPIT) and the empty Viaskin® (Sham) were applied on the back of anaesthetized mice. The skin was previously tape-stripped using scotch-tape for 10 times, changing each time the adhesive tape, for only one treated group (stripping+EPIT).

**Figure 1:**
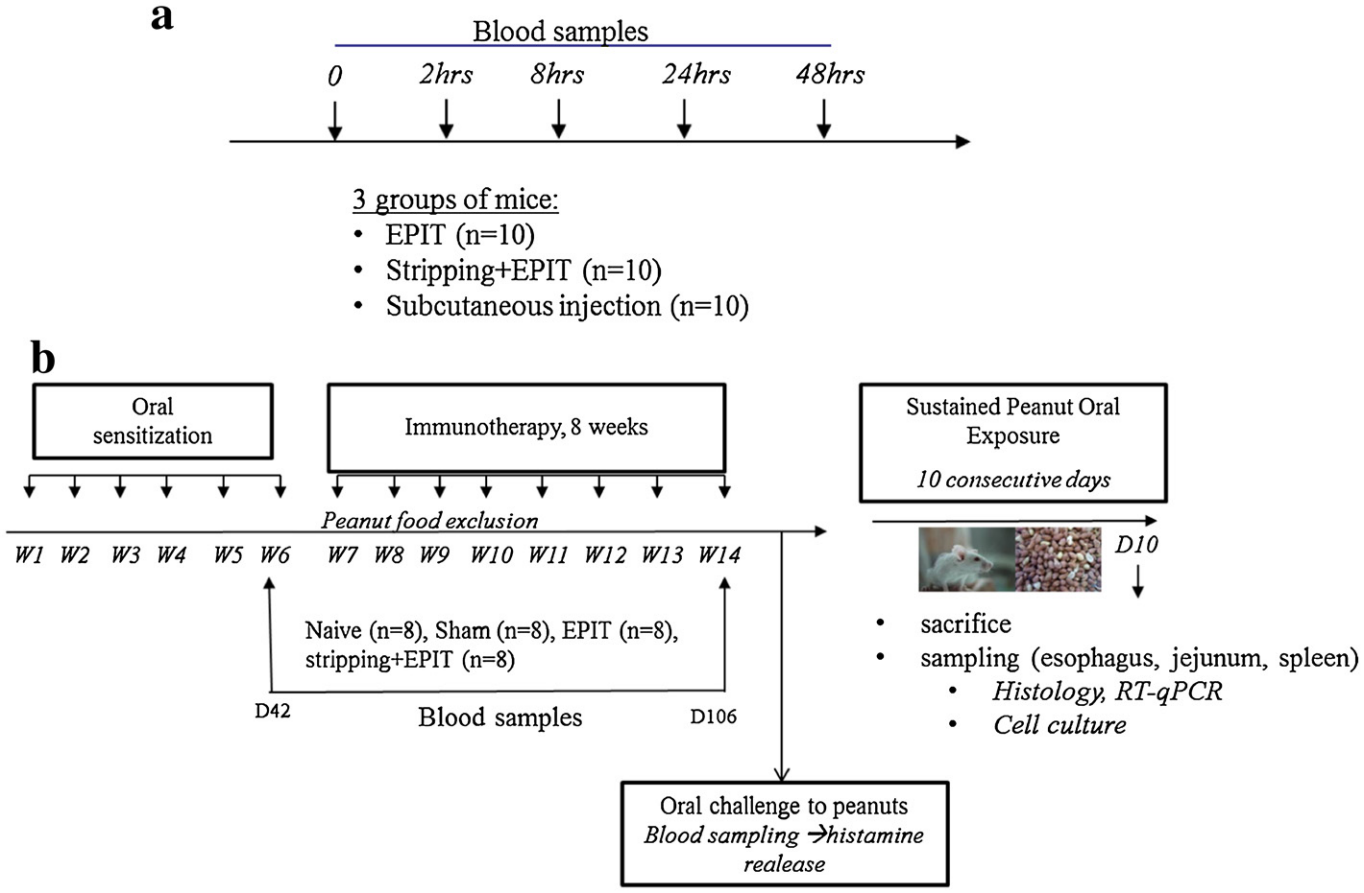
**a-Study design for the evaluation of peanut protein passage into blood stream after epicutaneous application on intact or stripped skin.** Naive mice were divided into 3 groups (n=10 for each). One group received a Viaskin® loaded with 500μg (Viaskin®-500) applied on intact skin (EPIT), another group received Viaskin®-500 applied on stripped skin (stripping+EPIT) and the last one received a subcutaneous injection containing 500μg of PPE. Blood samples were taken at different time points (0, 2, 8, 24, 48h) to quantify Ara h 1 in serum. **b- Study design for the sensitization of mice to peanuts proteins and evaluation of the effect by EPIT on intact or stripped skin on the induction of digestive lesions on esophagus and jejunum.** Twenty-four mice were sensitized to peanut proteins. Then, epicutaneous immunotherapy was conducted for 8 weeks in 8 sensitized mice on intact skin (EPIT), or in 8 sensitized mice on stripped skin (stripping+EPIT) and 8 other sensitized mice received a Sham treatment (Sham). After an oral challenge with high amounts of peanut proteins, histamine release was measured in blood samples. After that, a peanut regimen for 10 days was given to sensitized and naive mice. Mice were sacrificed to analyze esophagus and jejunum samples by histology and RT-qPCR. Blood samples were taken before the beginning of immunotherapy and after the 8-week period of treatment to measure specific immunoglobulins (IgE, IgG1, IgG2a).

2/ Protocol for safety concern (Figure 1a)

In order to evaluate the free passage to bloodstream after epicutaneous administration of PPE by Viaskin®, naive mice were received a single application of Viaskin® loaded with 500μg of PPE for 48h (Viaskin-500). Subcutaneous injections (200μl containing 500μg of PPE) as positive control of bloodstream passage were done.

3/ Protocol for efficacy concern (Figure 1b)

EPIT was performed using the epicutaneous delivery system Viaskin® (DBV technologies, Paris France) and the treatment protocol which has been previously described [3,4]. Briefly, epicutaneous treatment on intact skin or stripped skin was done once a week for 48h during 8 consecutive weeks.

### Measurement of Ara h 1 in serum samples

Blood was collected by retro-orbital bleeding in empty tubes before the application of Viaskin-500 (t0) and 2h, 8h, 24h and 48h after the application of Viaskin-500. Tubes were centrifuged at 10000g for 10mn and then sera were stored at −20°C until used. Commercial ELISA kit (Indoor Biotechnologies) was used for the quantification of Ara h 1 in serum samples. Manufacturer’s instructions were adapted to a measurement in serum matrix (under the FDA 2001 guidelines). The limit of quantification was determined at 7.8ng/ml, ie. less than 0.06% of 500μg PPE loaded into Viaskin®.

### Measurement of plasma peanut-specific IgE, IgG1 and IgG2a

Blood was collected by retro-orbital bleeding using tubes containing EDTA, 10 days after the last intra-gastric administration and at the end of sustained peanut oral exposure. Plasma were stored at −20°C until used. Peanut specific IgE, IgG1 and IgG2a levels were determined by ELISA as described previously [3,4]. As the high level of IgG might lead to underestimation of the sIgE level, the ELISA method has been confirmed by a reverse enzyme allergo-sorbent assay (EAST).

### Measurement of histamine release in blood samples after oral challenge to peanuts

Histamine increase in blood reflects the percentage of mast cells degranulation [9]. It was assayed in plasma samples 30 minutes after peanut oral challenge as a marker of anaphylactic reaction. Mice of each group were challenged at 30-minute intervals by 2 oral administrations of 10 mg PPE diluted in 200 μl of PBS. Histamine was assayed using a competitive enzyme immunoassay kit (SPI-BIO, Montigny-le-Bretonneux, France) in blood collected 30 minutes after the second oral challenge.

### Determination of splenocyte cytokine profiles

Following the peanut oral exposure and immediately after sampling esophagus and jejunum segments, splenocytes from each group were prepared as described previously [2]. Cells were cultured in 24-well plates (2 x 10^6^/well/ml) in presence or absence of PPE (100μg/ml) or concanavalin A (10μg/ml, data not shown). Supernatants were collected after 72 hrs of culture and stored at −20°C until use. Cytokine levels were determined using Bioplex cytokine assay® (BioRad, Marnes-la-Coquette, France) according to the manufacturer’s instructions.

### Analysis of esophageal eosinophilia and jejunum villus atrophy

Esophagus and jejunum were collected and fixed in 4% neutral-buffered formalin and prepared for analysis as already described [5]. Three sections of esophagus and 6 sections of jejunum were analyzed in a double-blind manner. Eosinophils were counted by a skilled European College of Veterinary Pathologists (ECVP) -certified pathologist and results were expressed as number of eosinophils per mm^2^. The ratio of villous height to crypt depth was evaluated using 6 intermediate-powered fields randomly selected around the jejuna lumen.

### Modulation of cytokine mRNA expression into esophagus by EPIT

Total RNA from esophageal sections was sampled in RNAlater® and extracted using RNeasy Mini Kit (Qiagen, Courtaboeuf, France) as already described [5]. The murine primer sequences designed with the OLIGO6 software package were already described. Quantitative PCR analyses in real time were performed with the LightCycler®480 Real-Time PCR system using SYBR-green fluorescence (Roche Diagnostic, Mannheim, Germany) for quantification. Results were presented as mRNA expression in the naive, EPIT, stripping+EPIT and Sham animals. Target gene expression was calculated relative to the expression of βactin and SDHA in each experimental sample, using the ΔCq method. Each set of quantitative PCR reactions were also run with negative controls without RNA and without RT.

### Statistical analysis

The GraphPad Prism Software 5.02 (San Diego, CA, USA) was used for statistical analysis (n=8 mice per group). Results are expressed as mean ± standard deviation (SD). Antibody, cytokine and mRNA expression responses were analyzed using analysis of variance (ANOVA) and Tukey’s test for intergroup comparison. For histological analyses, statistical significance comparing different sets of mice was determined by Student’s *t* test.

## Results

### Safety of epicutaneous application on intact skin as opposed to stripped skin

**Figure 2:**
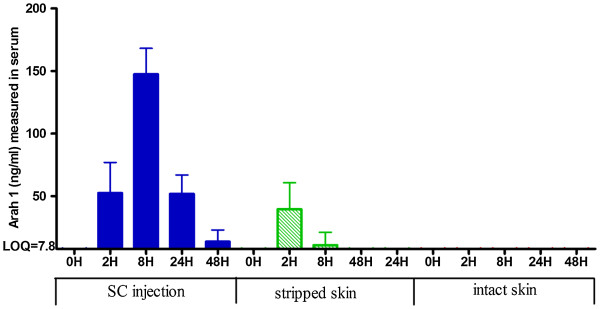
**Quantification of Ara h 1 in serum sample of mice.** Quantity of Ara h 1 was measured in serum samples after epicutaneous administration on intact or stripped skin or subcutaneous administration of 500μg of PPE. Results were expressed in ng/ml as means ± SD for each group.

In mice treated by subcutaneous injection of 500μg of PPE, serving as positive controls of delivery into the bloodstream, a high quantity of Ara h 1 was detected from 2h to 48h, with a peak at 8h (147.5 ± 20.6ng/ml) (Figure 2). When Viaskin®-500 was applied on intact skin, no Ara h 1 was detected in the serum from 0 to 48h. When Viaskin®-500 was applied on stripped skin, a limited quantity of Ara h 1 was detected in the serum at 2h (39.5 ± 21.2ng/ml) and 8h (10.8 ± 5.4ng/ml) after the application. For both EPIT groups, the quantity of PPE remaining inside the Viaskin® after 48h was measured at a similar level (25μg for EPIT and 20μg for stripping+EPIT, quantified by total protein assay) whereas the quantity transferred into the skin (epidermis and dermis) was a little higher at 2h and 8h for the intact skin group (data not shown, 1007ng/ml and 388ng/ml for EPIT vs 677ng/ml and 146ng/ml for stripping+EPIT).

### Modulation of humoral/cellular responses by EPIT depending on the integrity of epidermis

**Figure 3:**
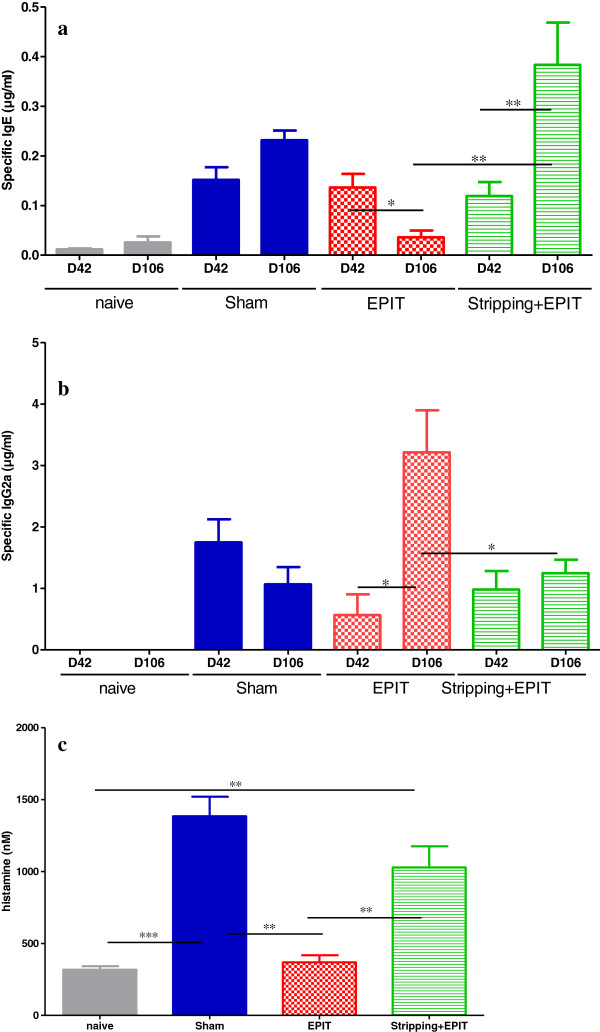
**Systemic responses induced in mice after oral sensitization and epicutaneous immunotherapy (a) Quantity of specific IgE and (b) specific IgG2a expressed in μg/ml.** Data are expressed as means ± SD for each group, D42 after oral sensitization, D106 after immunotherapy and sustained peanut exposure. (**c**) Measurement of histamine release in bloodstream after oral challenge to peanuts. Data are expressed as means ± SD for each group. * p<0.05, ** p<0.01, *** p<0.001.

The serological responses were analyzed after both sensitization (D42) and a 8-week EPIT (D106) (Figure 3). No specific antibodies to PPE were detected for naive mice. In the sham group, specific IgE increased significantly after sensitization and were maintained during 8-week of treatment, with no modification of specific IgG2a. When EPIT was applied on intact skin, specific IgE decreased from D42 to D106 (from 0.14 to 0.04 μg/ml, p<0.05) and specific IgG2a increased (from 0.56 to 3.21 μg/ml, p<0.05). To the opposite, when EPIT was applied on stripped skin, specific IgE increased (from 0.12 to 0.38 μg/ml, p<0.01) and specific IgG2a were not modified (0.98 vs 1.25 μg/ml, ns). The IgG1/IgG2a ratio significantly differed between EPIT and Sham or stripping+EPIT (respectively, 18 vs 228 or 227, p<0.001).

Levels of histamine released in plasma sampled 30 min after oral challenge were higher in sham (1384 nM) than in naive mice (317 nM, p<0.001). It was significantly reduced by EPIT done in intact skin (369 nM, p<0.01 vs. sham) whereas the release was still high for mice treated by EPIT applied on stripped skin (1028 nM, p<0.01 vs naive and EPIT).

**Figure 4:**
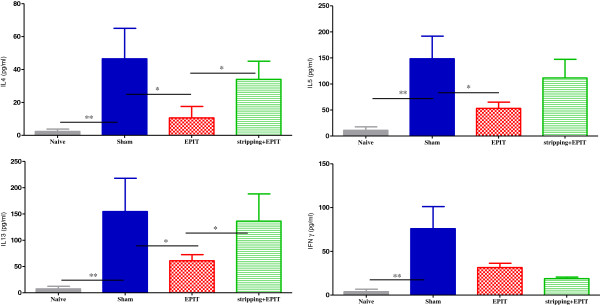
**Cellular responses induced in mice after oral sensitization and epicutaneous immunotherapy.** Measurement of Th2 cytokine levels (IL-4, IL-5, IL-13) and IFN-γ secretion by splenocytes collected from each group of mice (naive, Sham, EPIT, stripping-EPIT) immediately after sacrifice. Splenocytes were prepared and stimulated with PPE for 72 hrs. Cytokines were measured by Bioplex cytokine assay®. Data are presented as means ± SD for each group, * p<0.05, ** p<0.01.

Splenocytes were reactivated in vitro in presence of PPE. In sham mice, they specifically secreted Th1 and mainly Th2 cytokines in comparison to naive mice (Figure 4): IL-4 (46.5 vs 2.4 pg/ml, p<0.01), IL-5 (148.3 vs 11.0, p<0.01), IL-13 (154.6 vs 7.3, p<0.01) and IFN-γ (75.9 vs 3.9, p<0.01). When mice were treated by EPIT on intact skin, Th2 cytokines decreased: IL-4 (10.6 pg/ml vs sham at 46.5 pg/ml, p<0.05), IL-5 (53.1 pg/ml vs sham at 148.3 pg/ml, p<0.05), IL-13 (60.9 pg/ml vs sham at 154.6 pg/ml, p<0.05) and IFN-γ (31.4 pg/ml vs sham at 75.9 pg/ml, ns). To the opposite, when EPIT was applied on stripped skin, the secretion of Th2 cytokines was maintained and the Th1 pathway was down regulated: IL-4 (33.9 pg/ml vs sham, ns), IL-5 (111.5 pg/ml vs sham, ns), IL-13 (136.3 pg/ml vs sham, ns) and IFN-γ (18.8 pg/ml vs sham, ns). No cytokine secretion was detected by stimulation in medium alone.

### Assessment of esophageal and jejunal lesions

**Figure 5:**
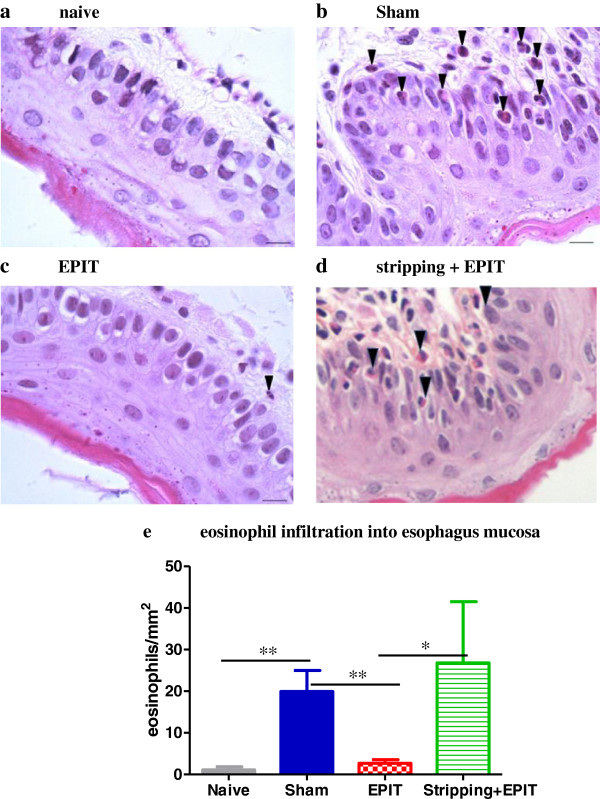
**Effect of EPIT on intact or stripped skin on the induction of injuries in esophagus.** Microscopic analysis of eosinophils in the esophagus at 100x high-powered fields (**a**-**d**). Most eosinophils are located in the lamina propria, submucosa and epithelial layer of the Sham and stripping-EPIT groups and to a lesser extend of the EPIT group. A difference in the thickness of epithelium is observed between naïve/EPIT and Sham/stripping+EPIT. (**e**) For eosinophils, the results are expressed as number of eosinophils per mm^2^ and data are presented as means ± SD for each group, * p<0.05, ** p<0.01.

Naive mice exposed to a peanut exclusive diet for 10 days did not exhibit any esophagus injuries (Figure 5a). In the sham group, the esophagus showed a massive infiltration with inflammatory cells, particularly eosinophils, in the lamina propria around the vascular plexus or more diffusely in the most severe cases (Figure 5b). When EPIT was applied on intact skin (Figure 5c), the tissue sections following sustained peanut food exposure exhibited lower cell infiltration in the lamina propria and epithelium than in sham, with an aspect similar to naive mice. When EPIT was applied on stripped skin (Figure 5d), the aspect was similar to that of sham mice. The eosinophil infiltration in the esophagus of sham mice (Figure 5e) was significantly higher than in naive mice (20 eosinophils/mm^2^ vs 1 eosinophil/mm^2^, p<0.01), clearly smaller in EPIT than in sham (3 eosinophils/mm^2^, p<0.01). For EPIT on stripped skin, the high infiltration of eosinophils was maintained (27 eosinophils/mm^2)^, similar to sham and higher than in EPIT on intact skin (p<0.05).

**Figure 6:**
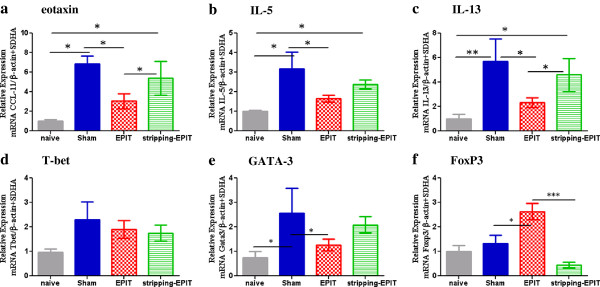
**Effect of EPIT on intact or stripped skin on the mRNA expression of cytokines and transcription factors in esophagus mucosa.** Cytokine mRNA from esophagus segments collected 24 hrs after stopping peanut diet was assayed by RT-qPCR. Results are presented as mRNA expression of naïve, Sham, or EPIT animals. The relative levels of gene expression were calculated by reference to mRNA levels of SDHA and β-actin in each sample. (**a**) eotaxin, (**b**) IL-5, (**c**) IL-13, (**d**) T-bet, (**e**) GATA-3, (**f**) FoxP3. * p<0.05, ** p<0.01 , *** p<0.001.

At the molecular level, esinophil infiltration was accompanied by an increased esophageal expression of eotaxin, IL-5, IL-13, GATA-3 and Tbet mRNA for Sham group (Figure 6). EPIT on intact skin reduced the expression of Th2 cytokines as indicated by significantly lower mRNA levels vs sham for eotaxin, IL-5, IL-13 and GATA-3 (p<0.05) and had no effect on Tbet. The expression of FoxP3 was significantly higher after EPIT compared with the sham and naive groups (p<0.05). When EPIT was applied on stripped skin, Th2 cytokine (eotaxin, IL-5, IL-13, GATA-3) mRNA levels were similar to those obtained for sham group and no induction of FoxP3 mRNA was observed in comparison to EPIT on intact skin (respectively, 0.5 vs 2.6, p<0.001).

**Figure 7:**
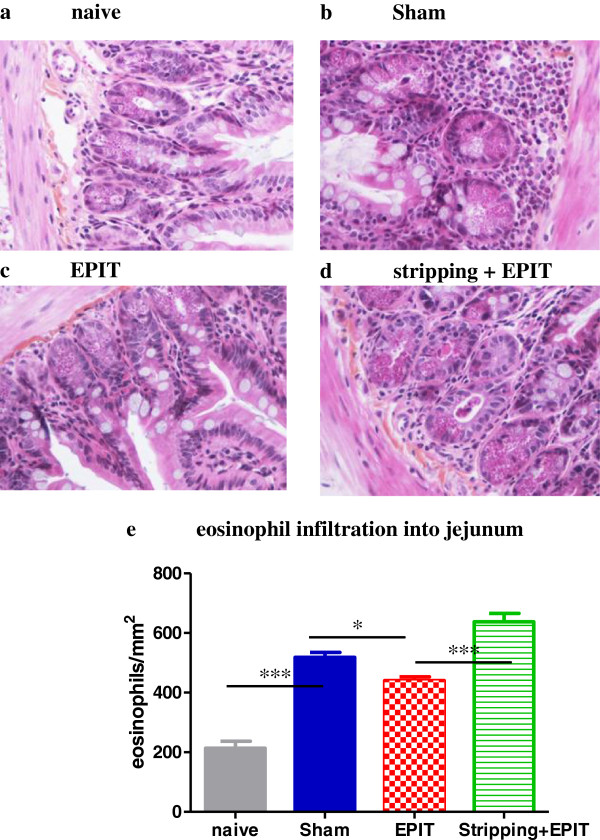
**Effect of EPIT on intact or stripped skin on the induction of jejunal lesions jejunum segments collected and analyzed by microscopy after HES coloration (x40).** (**a**-**d**) Inflammatory infiltration, particularly of eosinophils is shown. (**e**) quantification of the eosinophilic infiltration into jejunal mucosa. The results are expressed as number of eosinophils per mm^2^ and data are presented as means ± SD for each group, * p<0.05, *** p<0.001.

In the jejunum, sustained oral exposure to peanuts was associated with obvious jejunal lesions (Figure 7). Compared with naive mice, the recruitment of inflammatory cells in the lamina propria consisted mostly of eosinophils in Sham group (Figures 7a and 7b), this infiltration was quantified to 519 eosinophils/mm^2^ vs 214 eosinophils/mm^2^ into naive mice (p<0.001). After EPIT on intact skin, the tissue sections of the jejunum obtained following the peanut exclusive diet showed a sub-mucosal eosinophilic infiltration that was reduced compared with sham (440 eosinophils/mm^2^ vs 519 eosinophils/mm^2^, p<0.05). When EPIT was done on stripped skin, the eosinophilic infiltration of the jejunum was maintained at a similar level than Sham group (638 eosinophils/mm^2^).

**Figure 8:**
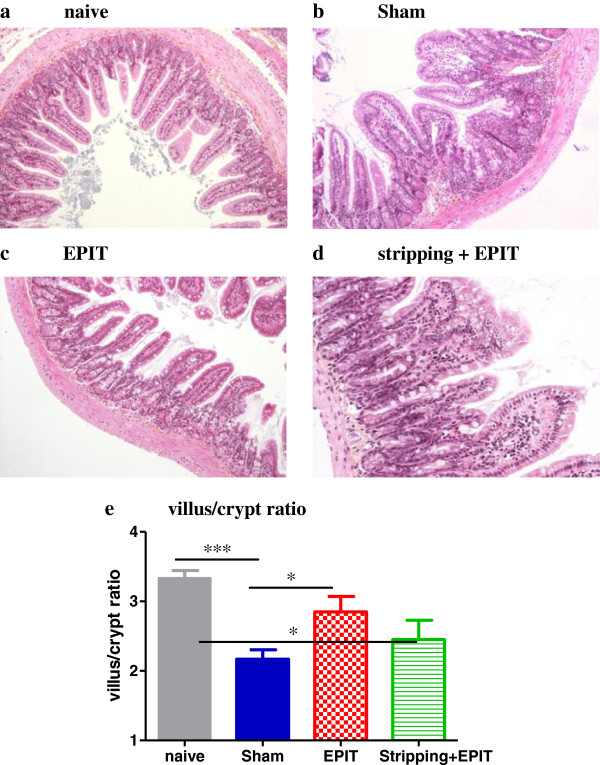
**Effect of EPIT on intact or stripped skin on the induction of villus sub-atrophia.** Measurement of the ratio of the villous height by crypt depth for each group under 10x high powered fields. Results are expressed as means ± SD, * p<0.05, *** p<0.001.

The sustained oral exposure to peanuts induced a degree of villus sub-atrophy (Figure 8) with, in the sham group, an overall decrease in villous height and increase in crypt depth, which significantly decreased the villus/crypt ratio compared with naive mice (2.2 vs 3.4, p<0.001). EPIT on intact skin prevented the modification of the villus height and the crypt depth observed with sham: the villus/crypt ratio did not decrease (2.9, p<0.05 vs Sham), and was similar to that in naive mice. When EPIT was applied on stripped skin, the aspect of villi and the villus/crypt ratio was similar to that of sham group, a (2.4, p<0.05 vs naive).

## Discussion

This study suggests that EPIT using Viaskin® is efficient only when applied on intact skin.

In the present study, the immune response generated by Viaskin® appears to be strongly influenced by the condition of the skin. When Viaskin® has been applied on intact skin, the profile of the immune response generated by the treatment is predominantly Th1/Treg whereas in case of Viaskin® applied on stripped skin, it is clearly Th2 oriented. The current work strongly suggests that during EPIT, skin preparation, ie. removing the stratum corneum layer and damaging epidermis, dramatically alters the outcome of treatment and immune reaction.

Skin stripping has never been investigated during the course of EPIT, but has long been considered an enhancing factor of sensitization. Tape-stripping in mice was shown to act as a natural adjuvant. Indeed, according to Strid et al. [10-12] and Spergel et al. [13], the application of antigen wihout adjuvant on previously stripped skin in naive mice switches the antigen-specific T helper cell responses from Th1-type to Th2-type: epicutaneous immunization on stripped skin converts an established Th1 response (induced by previous subcutaneous injection with adjuvant) into a Th2 response, with a specific reduction of IFN-γ and IgG2a and the enhancement of IL-4 and IgE. In model of food allergy in which mice were sensitized by epicutaneous application of ovalbumin on skin abraded by tape-stripping [14], mice developed diarrhea and accumulated mast cells in the small intestine, while vast amounts of MMCP-1were released from these cells into the circulation.

In vaccination models, a strong immune reaction was induced by application of antigen on the skin together with an adjuvant [6,7,15]. In these models, antigen and adjuvant were applied on the skin previously prepared by gentle removal of the superficial layer of the stratum corneum in order to enhance the transcutaneous passage of both the antigen and adjuvant. The immune responses were clearly Th2 oriented and results consistent with those reported here.

The importance of the state of skin in the profile of immune response on contact with antigen has also been illustrated in humans by Lack et al. [16], showing that the exposure of skin to peanut proteins may facilitate the sensitization process in very young children, when the normal architecture of the skin is altered by local or generalized eczema [16-18].

In our experiments, EPIT induced on intact skin a major decrease in specific IgE together with a huge increase in specific IgG2a whereas on stripped skin it reinforced specific IgE and did not modify specific IgG2a. The opposite modulation of humoral response was illustrated by the IgG1/IgG2a ratio that slightly decreased with EPIT on intact skin and significantly increased with EPIT on stripped skin. Moreover, at a systemic level, after oral challenge, histamine release was lower when mice were treated by EPIT on intact skin than on stripped skin. At the cellular level, splenocytes of EPIT group secreted lower levels of Th2 cytokines than sensitized and untreated mice.

However, in clinical situations, stripping of the skin appears not to be playing the same “clear-cut” role. Indeed, mouse skin is more sensitive to tape-stripping than human skin. In a recent paper, Senti et al. [8,19] treated patients allergic to pollen by repeated applications of pollen extract on a previously stripped skin with encouraging results. Despite no improvement in the provocation test, the primary outcome, in the active group versus the control group always showed a significant improvement of the seasonal symptoms (hay fever). The patch was applied for 48 hours on the skin prepared by 6 times tape-stripping.

In this study, we showed that EPIT on stripped skin leads to a free passage of allergens (ie. Ara h 1) into the bloodstream whereas no detectable level is measured when EPIT was applied on intact skin. The kinetics of allergen delivery is different: application on stripped skin induces a passive passage of allergens through the skin into the lymphatics, with high counts in the dermis and numerous allergen-positive cells in draining LNs as early as 2h after application [20] (paper in preparation). Dendritic cells targeted by the two modes of application of EPIT (intact vs stripped skin) exhibit different phenotypes in term of activation and maturation [20] (paper in preparation).Taken together - allergen specific capture by DCs through LNs and no detectable level of allergen in bloodstream - these results underline the safety of application only on intact skin. Importantly, these data are consistent with clinical observations. In the human trial, some local adverse events (33% of patients) and systemic allergic reactions required intervention (8% of patients) during the dose-effect study [19]. In children severely allergic to cow’s milk treated by EPIT on intact skin, no severe adverse event was reported [21].

The application on stripped skin clearly allows the passive and massive passage of allergen through the skin into the lymphatics [2] completely modifying the biodistribution of allergen and the targeted cells, i.e. less activated Langerhans cells. Tape-stripping also triggers mechanical injuries which activate keratinocytes and upregulate thymic stromal lymphopoietin (TSLP) expression by keratinocytes and mRNA expression of inflammatory cytokines, all of them involved in the polarization of skin DCs to elicit a Th2 response seeing that a link between TSLP expression and the pathogenesis of AD has been shown in several mouse models [22-24]. By contrast, in a recent paper, Li et al. (2012) describe an epicutaneous treatment on intact skin to prevent oral food sensitization in a mouse model [25]. More specifically, the authors showed that high-dose PPE cutaneous application on intact skin is capable of promoting skin local regulatory T-cell responses. At a systemic level, their results showed that the defined exposure of food allergens to intact skin suppresses the subsequent food allergy oral sensitization with suppression of multiple Ig isotypes (IgE, IgG1, IgG2a). Altogether, this greatly suggests that for treatment of Th2 disease, such as immunotherapy of food allergy, avoiding tape-stripping could be of importance, leading to efficacy of EPIT and to maintain the safety of the treatment. The mechanisms involved with the epicutaneous allergen application for the treatment of food allergy are being explored (data submitted for publication) and is likely due to a specific targeting to Langherans cells responsible to antigen presentation to T cells in lymph nodes and Treg expansion [26].

The model of peanut-sensitized mice exposed to sustained peanut oral regimen in order to induce esophageal and jejunum injuries was recently published [5]. The digestive tract is one of the main organs targeted during food allergies. Based on our previous model developed for the evaluation of new therapeutics in the field of food allergies [5], we compared the eosinophilic infiltration in mice treated by EPIT on intact or stripped skin. As already published, the decrease of the digestive eosinophil infiltration induced by the ingestion of peanut in sensitized mice treated by EPIT on intact skin was accompanied by a significant decrease of mRNA expression of Th2 cytokines, eotaxin and GATA-3 as well as an increase of FoxP3, underlining the involvement of Tregs in down-regulation of the Th2 pathway. EPIT on stripped skin maintain the high infiltration of eosinophil in jejunum mucosa as well as the villus sub-atrophia, did not induce any increase in mRNA expression of FoxP3 and maintain the high mRNA expression of Th2 cytokines and GATA-3.

## Conclusion

In conclusion, epicutaneous immunotherapy through repeated applications of allergen should be performed on normal, non-inflamed skin, ie. intact skin, in order to insure the safety of the treatment by avoiding a massive free passage of the allergen into the blood stream but also to induce a tolerogenic immune profile.

## Abbreviations

DCs: Dendritic Cells; EPIT: Epicutaneous Immunotherapy; Ig (E: G1, G2a), Immunoglobulin type E, G1, G2a; RT-qPCR: Reverse Transcriptase-quantitative-Polymerase Chain Reaction; Th2 cells: T helper type 2 cells.

## Competing interests

This study was supported by DBV Technologies, the developer and owner of Viaskin®. Lucie Mondoulet, Vincent Dioszeghy, Emilie Puteaux, Mélanie Ligouis, and Veronique Dhelft are DBV Technologies employees.

Franck Letourneur, Christophe Dupont and Pierre-Henri Benhamou received honoraria and/or compensation in regards to the study, as investigators, coordinators or experts, in relation with the time spent on the study. The authors declared that they have no competing interests.

## Authors' contributions

LM, CD, PB conceived and designed the experiments. LM, VDi, EP, ML, VDh and FL performed the experiments. LM, EP, ML and VDh analyzed the data. LM, CD and PB drafted the manuscript. All authors read and approved the final manuscript.
